# Reconsidering immunotherapy resistance: the emerging role of the tumor microbiome in head and neck and lung cancers

**DOI:** 10.1097/MS9.0000000000004902

**Published:** 2026-04-14

**Authors:** Sarum Ali Khan, Muhammad Adnan Qamar, Taimoor Ali, Muhammad Hassan Omer, Aiman Tahir

**Affiliations:** a Department of Medicine, Wah Medical College, Wah Cantt, Pakistan; b Department of Internal Medicine, Spinghar Medical University, Kabul, Afghanistan; c Department of Medicine, Azad Jammu and Kashmir Medical College, Muzaffarabad, AJK, Pakistan

**Keywords:** Fusobacterium nucleatum, gut–tumor axis, head and neck squamous cell carcinoma, immune checkpoint inhibitors, immunotherapy resistance, intratumoral microbiome

## Abstract

Immunotherapy with immune checkpoint inhibitors (ICIs) has revolutionized treatment for non-small cell lung cancer (NSCLC) and head and neck squamous cell carcinoma (HNSCC), yet resistance limits durable responses in many patients. Emerging evidence implicates the intratumoral microbiome – comprising bacteria, fungi, and viruses within tumor tissues – as a key modulator of tumor biology, immune infiltration, and ICI sensitivity, beyond traditional tumor-intrinsic and immune factors. In HNSCC, human papillomavirus (HPV)-negative tumors exhibit higher oncobacteria abundance than HPV-positive ones, with elevated levels linked to worse survival in HPV-positive oropharyngeal cases, suggesting an immunosuppressive tumor microenvironment that may influence ICI outcomes. In NSCLC, intratumoral taxa such as *Fusobacterium nucleatum* and *Bacteroides fragilis* promote progression and evasion via immune checkpoint modulation (PD-1/PD-L1), pro-inflammatory pathways (toll-like receptors and cytokines like interleukin-6/tumour necrosis factor-alpha), metabolic reprogramming (PI3K/AKT), and recruitment of suppressive cells (neutrophils and myeloid-derived suppressor cells). Pan-cancer studies show microbial enrichments and compositional shifts in responders versus non-responders to ICI, with metabolites (e.g., lactate and succinic acid) driving M2 macrophage polarization, T-cell suppression, and resistance. The gut–tumor axis further exacerbates refractoriness through systemic dysbiosis and immune alterations. Preclinical models indicate that targeted microbiome interventions – such as fecal microbiota transplantation, specific probiotics (e.g., *Bifidobacterium* spp. and *Akkermansia muciniphila*), or selective antibiotics – can restore antitumor immunity, enhance ICI efficacy, and minimize broad dysbiosis risks. Integrating intratumoral microbial profiling into HNSCC and NSCLC clinical trials could refine patient stratification, uncover predictive biomarkers, and accelerate microbiome-directed adjunct therapies, advancing precision oncology and expanding immunotherapy benefits.

*To the Editor*,

This manuscript is in accordance with the TITAN checklist guidelines 2025 ^[^1^]^.

Although immunotherapy has revolutionized the treatment of solid tumors like non-small cell lung cancer (NSCLC) and head and neck squamous cell carcinoma (HNSCC), resistance still limits long-term benefits for a significant percentage of patients. Conventional explanations focus on immune dynamics and tumor genetics, but growing evidence points to the intratumoral microbiome as an important but little-known factor. Tumor microenvironment, immune infiltration, and treatment sensitivity are all actively modulated by this microbial community, which includes bacteria, fungi, and viruses. This provides new insights into overcoming resistance.

In HNSCC, distinct microbial patterns correlate with tumor characteristics. Human papillomavirus (HPV)-negative tumors harbor higher oncobacterial abundance compared to HPV-positive tumors, a difference that persists within oropharyngeal subsites (*P* = 1.9 × 10^−2^). Notably, increased oncobacterial burden is associated with poorer overall and progression-free survival in HPV-positive oropharyngeal cases, even after stage adjustment. These findings suggest that microbial composition may contribute to an immunosuppressive microenvironment and influence treatment responsiveness, although mechanistic validation is ongoing^[^2^]^.

Beyond HNSCC, similar microbiome–tumor interactions have been observed in other solid malignancies, underscoring that this phenomenon is not site-restricted, particularly in lung cancer. Compared with adjacent normal tissue, tumors demonstrate enrichment of taxa such as Firmicutes, Proteobacteria, Bacteroidetes, and oral-associated genera, including *Prevotella, Streptococcus*, and *Veillonella*. Certain species, notably *Fusobacterium nucleatum* and *Bacteroides fragilis*, have been associated with immune evasion and adverse outcomes.

Mechanistically, microbial communities may influence immune checkpoint signaling (PD-1/PD-L1 and CTLA-4), activate pattern recognition receptors, and sustain pro-inflammatory cytokine production, fostering a chronically dysregulated immune milieu. They can also reshape tumor metabolism and macrophage polarization, promote suppressive myeloid cell recruitment, and activate oncogenic pathways such as PI3K/AKT and NF-κB. Experimental models demonstrate that microbial depletion can attenuate tumor growth, while specific taxa – particularly *F. nucleatum* – have been linked to enhanced metastatic potential and resistance to immune checkpoint blockade. Collectively, these findings support a role for intratumoral microbiota in modulating immunotherapy responsiveness^[^3^]^. Pan-cancer analyses of metastatic tumors have identified associations between specific microbes and therapeutic outcomes, including in lung cancers, where certain enrichments are observed in relation to response patterns. Longitudinal sampling has revealed shifts in microbial composition between responders and non-responders to immune checkpoint blockade^[^4^]^. Intratumoral microbes and their metabolites mechanistically influence both progression and treatment. Resistance can be fostered through the activation of oncogenic pathways, alterations in autophagy, the disruption of apoptosis, and the inhibition of DNA repair mechanisms^[^5^]^. Macrophages can be induced to adopt an M2 phenotype by short-chain fatty acids and other metabolites, thereby suppressing anti-tumor immunity and facilitating metastasis. Inconsistent responses to immune checkpoint inhibitors (ICIs) are attributable to diminished T-cell activity and heightened checkpoint expression^[^6^]^. Furthermore, the gut–tumor microbiome axis adds complexity, as intestinal dysbiosis can indirectly contribute to resistance via systemic immune alterations. Pathogenic shifts in the gut, which metabolize medications or release vesicles that promote distant tumor growth, may intensify refractoriness in patients receiving ICIs^[^7^]^. Promising interventions include selective antibiotics, probiotics, engineered microbes, or fecal microbiota transplantation (FMT) to restore microbial balance and enhance tumor responsiveness to ICIs. Preclinical models have demonstrated that targeted modulation – such as FMT from ICI responders, supplementation with specific strains (e.g., *Bifidobacterium* spp. and *Akkermansia muciniphila*), or probiotics – can improve ICI efficacy by promoting antitumor immunity (e.g., enhanced CD8+ T-cell infiltration and cytokine production) while minimizing broad dysbiosis risks, as shown in murine models of melanoma, lung cancer, and other tumors where such approaches reduced tumor growth or restored responses in antibiotic-disrupted microbiomes^[^3,7^]^.

In the end, the tumor microbiome is a key area for figuring out why immunotherapy does not work. Incorporating microbial profiling into trials for HNSCC and NSCLC could improve patient selection and inspire microbiome-directed adjuncts. This holistic approach has the potential to make precision oncology more inclusive and impactful, benefiting a wider range of patients.

## Data Availability

All data used are accessible.

## References

[1] Transparency In The reporting of Artificial INtelligence – the TITAN guideline. Premier Science 2025. Accessed 2025 December 4. https://premierscience.com/pjs-25-950/.

[2] Kerr TD Silver NL Duggal R. HPV status impacts oncobacteria abundance and prognostic relevance in head and neck squamous cell carcinoma. Oncogene 2025:44:2217–23. 40494952 10.1038/s41388-025-03463-4 PMC12183080

[3] Zhao Y Yang Z Wu D. Dissecting the intratumoral microbiome landscape in lung cancer. Front Immunol 2025;16:1614731. 40777004 10.3389/fimmu.2025.1614731 PMC12328415

[4] Battaglia TW Mimpen IL Traets JJH. A pan-cancer analysis of the microbiome in metastatic cancer. Cell 2024:187:2324–2335. 38599211 10.1016/j.cell.2024.03.021

[5] Ciernikova S Sevcikova A Mego M. Targeting the gut and tumor microbiome in cancer treatment resistance. Am J Physiol Cell Physiol 2024; 327:C1433–50. 39437444 10.1152/ajpcell.00201.2024

[6] Zhai Z Li X Shang S. Intratumoral microbiota and metabolites: dual roles in cancer progression and therapeutic opportunities. Cell Commun Signal 2026;24:80. 41484626 10.1186/s12964-025-02623-z PMC12870031

[7] Yang L Wang Q He L. The critical role of tumor microbiome in cancer immunotherapy. Cancer Biol Ther 2024;25:2301801. 38241173 10.1080/15384047.2024.2301801 PMC10802201

